# Histological and Clinical Characteristics of Patients with Chronic Hepatitis C and Persistently Normal Alanine Aminotransferase Levels

**DOI:** 10.1155/2014/760943

**Published:** 2014-05-07

**Authors:** Bakht Roshan, Grace Guzman

**Affiliations:** ^1^Department of Medicine, Section of Hepatology, University of Illinois at Chicago, 840 South Wood Street, Suite 130, Chicago, IL 60612, USA; ^2^Department of Pathology (MC847), University of Illinois at Chicago, 840 South Wood Street, Suite 130, Chicago, IL 60612, USA

## Abstract

Patients with chronic hepatitis C virus (HCV) infection and persistently normal alanine aminotransferase (PNALT) are generally described to have mild liver disease. The aim of this study was to compare clinical and histological features in HCV-infected patients with PNALT and elevated ALT. Patients presenting to the University of Illinois Medical Center, Chicago, who had biopsy proven HCV, an ALT measurement at the time of liver biopsy, at least one additional ALT measurement over the next 12 months, and liver biopsy slides available for review were identified. PNALT was defined as ALT ≤ 30 on at least 2 different occasions over 12 months. Of 1200 patients with HCV, 243 met the study criteria. 13% (32/243) of patients had PNALT while 87% (211/243) had elevated ALT. Significantly more patients with PNALT had advanced fibrosis (F3 and F4) compared to those with elevated ALT (*P* = 0.007). There was no significant difference in the histology activity index score as well as mean inflammatory score between the two groups. In conclusion, in a well-characterized cohort of patients at a tertiary medical center, PNALT did not distinguish patients with mild liver disease.

## 1. Introduction

Hepatitis C virus (HCV) infection is reported to have a prevalence of approximately 3% worldwide [1]. Almost 80% of those infected go on to develop chronic infection. Majority of patients with chronic HCV have a mild, asymptomatic elevation in serum transaminase levels with no significant clinical symptoms. Around 25% of patients with chronic HCV have persistently normal alanine aminotransferase (PNALT) [2].

Definition of normal alanine aminotransferase (ALT) has changed over time and reference range for normal ALT differs based on different laboratory cutoffs. Prati et al. [3] in 2002 suggested new cutoffs with 30 U/L (international unit) for men and 19 U/L for women compared to 40 U/L and 30 U/L for men and women, respectively. This resulted in improved sensitivity but decreased specificity. Similarly, definition of PNALT differs widely. A 2009 American Association for the Study of Liver Disease (AASLD) practice guideline suggested an ALT value of 40 U/L on 2-3 different occasions separated by at least a month over a period of 6 months [4]. Others have used 3 different ALT levels equal to or below upper limit of normal (ULN) separated by at least 1 month and sometimes over a period of 18 months [5]. Thus, there is no consensus on a universal definition of PNALT.

It was generally thought that people with PNALT have a mild liver disease and the degree of liver fibrosis is minimal [6–14]. Based on this, people with PNALT were initially monitored conservatively without treatment. Later on, it was realized that a considerable number of such patients developed significant inflammation and fibrosis over time [15]. More recently, treatment has been recommended along the same lines for patients with PNALT as patients with elevated ALT [4].

Although more data is becoming available about the relationship of liver enzymes and course of chronic HCV infection, data regarding HCV infection and PNALT is relatively scarce. Because of variation in the definition of PNALT, fewer studies have looked at the relationship of PNALT with chronic HCV infection using updated normal ALT definitions [16].

Department of Hepatology at the University of Illinois (U of I) medical center, Chicago, had a database of over 1200 patients with chronic HCV infection. Medical records of these patients were reviewed in an effort to characterize patients with chronic HCV infection and PNALT. Histological and clinical parameters for patients with PNALT as well as elevated ALT were analyzed.

## 2. Materials and Methods

Database of patients with HCV infection presenting to U of I medical center, Chicago, was reviewed. These patients had a liver biopsy done between 1996 and 2007. Patients with biopsy proven HCV infection and a detectable HCV ribonucleic acid (RNA) in blood were chosen. Of these, patients with an ALT at liver biopsy, at least one additional over the next 12 months, and liver biopsy slides available for review were identified.

Most of the liver biopsy procedures were done at U of I medical center and in cases where biopsies were done at outside facility they were read again at U of I medical center. Two expert hepatologists, who were masked to clinical data, assigned Knodell et al. [17] score to liver biopsies. Intervals for ALT measurement were chosen around the time of liver biopsy as well as 3, 6, and 12 months after biopsy. Patients with end-stage renal disease like those on dialysis and stage IV chronic kidney disease with creatinine clearance of 15–29, those who received organ transplant, those with co-infection with HIV, those who were positive for Hepatitis B surface antigen (HBsAg), and those receiving antiviral therapy for chronic HCV were excluded.

PNALT was defined as ALT ≤ 30 U/L on at least 2 different occasions over 12 months. Strict PNALT was defined as ALT ≤ 30 U/L for males and ≤19 U/L for females.

Demographic data including age at biopsy, gender, and race were recorded. Clinical data included body mass index (BMI), alcohol use, tobacco use, and presence of diabetes mellitus (DM). HCV virus was further characterized by recording HCV RNA levels, genotype, and duration of infection. Histological data included individual markers of inflammation like portal tract inflammation, piece meal necrosis, and lobular inflammation as well as fibrosis according to Knodell et al. scoring system. Inflammatory score (sum of portal tract inflammation, piece meal necrosis, and lobular inflammation) and histologic activity index (HAI) score (sum of inflammatory score and fibrosis) were calculated. Histologic data from PNALT was then compared with patients from elevated ALT group. Finally, clinical characteristics of PNALT with advanced fibrosis were compared with PNALT but with no advanced fibrosis.

Statistical analysis was performed using SPSS (SPSS Inc., Chicago, IL). Independent sample *t*-test and chi-squared test were used to calculate *P* values where appropriate.

## 3. Results

A total of 243 patients out of a database of 1200 patients with HCV satisfied the study criteria. Main reasons to exclude a large number of patients were a lack of detectable RNA despite biopsy report, outside biopsy report but slides not available for review, single or no ALT value, and patients undergoing treatments. Those analyzed were further divided into PNALT, strict PNALT, and elevated ALT group. 32 (13%) of these patients were identified as PNALT group and 211 (87%) were identified as elevated ALT group. Only 13 (5%) patients satisfied criterion for strict PNALT and this group was not analyzed further. The range of ALT values at different time intervals was specified (Table 1). 24 (75%) of PNALT patients were females while 85 (40%) with elevated ALT were females. 13 (41%) with PNALT were African American (AA) compared to 87 (41%) with elevated ALT, 14 (44%) were Caucasian (W) compared to 79 (38%) with elevated ALT, and 5 (15%) were Hispanic (H) compared to 44 (21%) with elevated ALT. There was no statistically significant difference in the racial distribution between PNALT and elevated ALT group.

There was a higher frequency of women in the PNALT group compared to the elevated ALT group (*P* = 0.001). Diabetes and alcohol use were more common among patients with elevated ALT compared to PNALT (*P* = 0.04 and 0.049, resp.). Most notably, patients with PNALT had a higher rate of cirrhosis (*P* = 0.007). There were no differences in age at biopsy, tobacco use, BMI, RNA level, and duration of infection between PNALT and elevated ALT groups (Table 2).

Further evaluation of liver histology showed no statistically significant difference in mean fibrosis score, mean portal tract inflammation score, mean piecemeal necrosis score (PMN), mean lobular inflammation score, mean histologic activity index (HAI) score, and mean inflammatory score between PNALT group and elevated ALT group (Table 3). Comparison of clinical characteristics of PNALT group with advanced fibrosis with PNALT group without advanced fibrosis showed that only platelet count was significantly different between the two groups (Table 4). Tables 5 and 6 characterize the distribution of HCV genotypes based on PNALT and HAI score, respectively.

## 4. Discussion

The natural history of chronic HCV infection with PNALT is poorly understood [18–20]. We attempt to describe the characteristics of patients with PNALT, which constitutes almost 25–30% of patients with chronic HCV infection. There are few significant findings from this work. Firstly, a high proportion of patients with PNALT had advanced fibrosis, and degree of inflammation was not significantly different than chronic HCV infection with abnormal ALT. Secondly, it was difficult to identify a substantially large set of patients with HCV infection and PNALT given that there is a significant fluctuation in the ALT level over time [9, 15]. Thirdly, patients with multiple comorbidities were excluded leaving a small cohort size.

We chose duration of 12 months to observe the levels of ALT instead of 6 months period. It is becoming clear that 6 months is probably too short given that in some cases ALT level may fluctuate after initial period of stability [7, 21–24]. Most patients with PNALT were females, which is consistent with earlier findings [7–9]. Abstinence from alcohol and lack of DM were associated with PNALT. There was no association with race. Similarly, age at biopsy, BMI, RNA level, and duration of infection were not significantly different between the two groups. HCV genotype distribution showed that a majority (81%) of patients belonged to genotype 1 and it is a well-characterized fact [25]. There was no significant difference in terms of distribution of genotypes between the 2 groups (Table 5). Also there was no significant difference in HAI according to genotype distribution (Table 6). HCV genotyping was performed in 181/243 (75%) patients and was missing in 62 (25%) patients. The likely reason was transition from paper to electronic records in 1990s and loss of some data.

Within PNALT, those with advanced fibrosis differed from those without advanced fibrosis by platelet count only. Other variables as shown in Table 4 did not achieve a significance level. Similarly, PNALT patients were divided based on low-normal ALT (<19) and high-normal ALT (20–30) for comparing HAI scores among them but no significance was seen (Table 7).

The most interesting finding was the comparison of histological data. Studies to date have been mentioning a milder disease for PNALT in terms of fibrosis and necroinflammation [7–9, 26–28]. Our study indicated that fibrosis and necroinflammation were comparable in both groups. Some studies have pointed to this fact as well [14, 29, 30]. This is an interesting finding given that despite significant inflammation (comparable to abnormal ALT) the ALT levels in some of these patients have been consistently low. The exact etiology of PNALT despite significant inflammation is not clear. Similarly, advanced fibrosis was more common in PNALT group as compared to the elevated ALT group (*P* = 0.007). It is thought that ALT levels normalize in patients with advanced fibrosis [31] and that is why some authors will advocate doing liver biopsy in patients with HCV infection and normal ALT levels [32]. It is interesting to note that the 6 patients with PNALT who had cirrhosis also had evidence of thrombocytopenia. Thrombocytopenia is a well-established marker of cirrhosis [33]. Our results indicate that platelet count can be used as a marker to predict fibrosis in patients with PNALT.

There were several limitations to this study. First, it was a retrospective study. Cases were excluded because only a single measurement was available. For instance, almost all patients in the study group had an ALT measured around biopsy but only slightly more than half had ALT measured around 12 months. This is why ALT was recorded around 3 months and 6 months as well. Second, sample size was relatively small and might not be a true representative of patients with PNALT. This might in particular be valid for PNALT with advanced fibrosis as 8 (25%) out of 32 patients with PNALT had F3-F4 while only 19 (9%) out of 211 patients with elevated ALT had F3-F4 (*P* = 0.007). It is not clear if the outcome would have been the same if denominator for PNALT was high.

Small sample size was caused mainly as described before as well as comorbid conditions like advanced kidney disease, HIV, HBsAg positive, and being on antiviral treatment. For example, 11 patients with PNALT were excluded as they had ESRD; ALT levels are known to be lower in ESRD [34, 35] secondary to an impaired immune response in patients with ESRD [36]. Third, ALT levels were measured at irregular intervals. This raises concern that those with PNALT and severe liver fibrosis may have been in biochemical remission. For example, of the 8 patients with severe liver fibrosis (stages 3 and 4) and PNALT, only 2 patients had 4 ALT measurements over 12 months (over the period of 0, 3, 6, and 12 months), while 3 patients had 3 ALT measurements over 12 months, and the remaining 3 patients had only 2 ALT measurements over the 12 months period. Thus, it is not possible to say with certainty that all patients with PNALT and severe liver damage had uniformly low ALT all along.

## 5. Conclusion

In conclusion, histological changes observed in HCV patients with PNALT will argue that ALT is not a reliable indicator of hepatic inflammation or fibrosis. In fact, PNALT was associated with advanced fibrosis in the current study. Female gender, absence of DM, and abstinence from alcohol were associated with PNALT. Platelet count could be used to predict fibrosis in patients with PNALT. These findings indicate the need for more studies with higher number of PNALT patients to look at the relationship of PNALT with changes occurring at histological and molecular levels.

## Figures and Tables

**Table 1:** ALT range expressed in international unit (U/L).

Interval	Biopsy	3 months	6 months	12 months
PNALT (median)	9–30 (24)	9–28 (20)	10–30 (22)	11–30 (21)
Elevated ALT (median)	16–987 (56)	14–57 (27)	12–1248 (45)	10–231 (43)

**Table 2:** Clinical data/distribution of patients.

	PNALT (*n* = 32)	Elevated ALT (*n* = 211)	Total (*n* = 243)	*P* value
Gender M/F	8/24	126/85	134/109	0.001
Race (W/AA/H)	14/13/5	79/87/44	93/100/49*	0.717
Alcohol (Y/N)	6/26	77/134	83/160	0.049
Tobacco (Y/N)	6/26	30/181	36/207	0.501
DM (Y/N)	2/30	46/165	48/195	0.04
Mean age at biopsy in years (*n*)	50 (31)	47 (211)	242*	0.153
Mean BMI (*n*)	26 (27)	25 (177)	204*	0.5
Mean RNA level in IU/mL (*n*)	1883693 (30)	4439614 (159)	189*	0.09
Duration of infection in years (*n*)	26 (21)	25 (152)	173*	0.768
Fibrosis (F0-1/F2-4)	10/22	84/127	94/149	0.354
Fibrosis (F0-2/F3-4)	24/8	192/19	216/27	0.007

*Data not available for all patients.

**Table 3:** Histological data.

	PNALT (mean ± SD)	Elevated ALT (mean ± SD)	*P* value
Fibrosis	2 ± 1	1.7 ± 1	0.067
Portal tract inflammation	1.66 ± 1	1.77 ± 1	0.46
PMN	1.47 ± 1	1.48 ± 1	0.96
Lobular inflammation	0.72 ± 1	0.9 ± 1	0.175
HAI score	6 ± 3	6 ± 3	0.94
Inflammatory score	4 ± 2	4 ± 2	0.7

**Table 4:** PNALT with advanced fibrosis versus PNALT without advanced fibrosis.

	PNALT with advanced fibrosis	PNALT without advanced fibrosis	Total number of patients *n* = 32	*P* value
Mean age at biopsy in years (*n*)	48 (8)	50 (23)	31*	0.7
ALT at biopsy in U/L (*n*)	23 (7)	21 (24)	31*	0.5
ALT at 12 months in U/L (*n*)	21 (4)	21 (19)	23*	0.88
Mean RNA level in IU (*n*)	134288 (6)	2321044 (24)	30*	0.127
Mean BMI (*n*)	27 (6)	26 (21)	27*	0.634
AST (*n*)	41 (7)	28 (24)	31*	0.061
Platelet count (*n*)	81000 (5)	257000 (21)	26*	0.001

*Data not available for all patients.

**Table 5:** HCV genotype characteristics.

Genotype	PNALT (*n*)	Elevated ALT (*n*)	Total (%)	*P* value
1	3	16	19 (11)	0.8
1a	10	60	70 (39)	
1b	8	49	57 (31)	
2a	1	5	6 (3)	
2b	0	13	13 (7)	
3a	1	13	14 (8)	
4	0	2	2 (1)	
Total	**23**	**158**	**181** (**100**)	

**Table 6:** HCV genotype and HAI score.

Genotype	HAI score			Total (%)	*P* value
	≤6	7–12	>13		
1	16	3	0	19 (11)	0.34
1a	45	25	0	70 (39)	
1b	38	16	3	57 (31)	
2a	4	2	0	6 (3)	
2b	6	7	0	13 (7)	
3a	9	5	0	14 (8)	
4	2	0	0	2 (1)	
Total (%)	**120 **(**66**)	**58 **(**32**)	**3 **(**2**)	**181 **(**100**)	

**Table 7:** ALT value and HAI score within PNALT.

	HAI score			Total	*P* value
	≤6	7–12	>13		
ALT value					
<19	7	1	0	8	0.4
20–30	15	8	1	24	
Total	**22**	**9**	**1**	**32**	
